# Integrating Community and Digital Support Through Social Prescribing to Improve Mental Health in Rural Older Adults in South Korea: Quasi-Experimental Study

**DOI:** 10.2196/76201

**Published:** 2026-05-05

**Authors:** Bo Zhao, Joo Aeh Lim, Subeen Lim, Hye Jin Yoon, Hae Kyung Kim, Seok Mi Hong, Xinyu Zhang, Eun Woo Nam

**Affiliations:** 1 Department of Health Services Management, School of Health Services Management, Anhui Medical University, Hefei, China; 2 Yonsei Global Health Center, Yonsei University, Wonju, Republic of Korea; 3 Gangwon Public Health Policy Institute, Wonju, Republic of Korea; 4 Graduate School of Health Science, Yonsei University, Wonju, Republic of Korea; 5 Saje Primary Health Care Post, Wonju, Republic of Korea; 6 Department of Health Administration, Graduate School, Yonsei University, 1 Yonseidae-gil, Wonju, Republic of Korea, 82-01038679569

**Keywords:** social prescribing, rural older adults, mental health, social support, cognitive function, community-based intervention, digital health, culturally tailored care.

## Abstract

**Background:**

Mental health issues such as depression, loneliness, and cognitive decline are prevalent among older adults. They are particularly pronounced in rural areas due to socioeconomic disadvantages, limited health literacy, and social isolation. These challenges have been exacerbated by the COVID-19 pandemic, highlighting the urgent need for accessible, community-based mental health strategies. Social prescribing—linking individuals to nonmedical community resources—offers a promising model, especially when coupled with culturally relevant and digitally facilitated interventions.

**Objective:**

This study aimed to evaluate the effectiveness of a pilot social prescribing project for individuals aged 65 years and older residing in rural South Korea. Specifically, it examined the changes in social support, loneliness, depression, cognitive function, and life satisfaction among the older adults before and after the intervention and the relationships among these factors.

**Methods:**

A quasi-experimental, nonequivalent control group pretest-posttest design was conducted with 294 participants from Wonju City, Gangwon-do (n=148 in the experimental group, n=146 in the control group). The 8-week intervention featured a community-based music storytelling program incorporating local cultural elements and digital education. Statistical analyses, including *t* tests, difference-in-differences analysis, and structural equation modeling, were conducted to evaluate changes in scores and the effectiveness of the program intervention.

**Results:**

The study confirmed that the social prescribing pilot project effectively reduced depression and increased social support and life satisfaction compared with the control group (*P*<.05, 95% CI). While cognitive function showed slight improvement, the change was not statistically significant. Social support emerged as a key mediating factor, positively influencing cognitive function and life satisfaction, and was negatively associated with depression. There was no statistically significant direct effect found between loneliness and cognitive function or social support.

**Conclusions:**

This pilot study supports the feasibility and mental health benefits of integrating community and digital support through culturally embedded social prescribing for rural older adults. The findings highlight the importance of leveraging local resources and social networks to address mental health disparities in underserved aging populations, offering valuable insights for policymakers and practitioners developing inclusive aging and health promotion strategies.

## Introduction

Developed countries like South Korea (hereafter “Korea”) are now experiencing super-aging speed and an increasing number of noncommunicable diseases with long-term conditions [1]. Well-known noncommunicable diseases such as cancer, diabetes, heart disease, Alzheimer disease, dementia, and mental health ailments pose devastating health consequences for individuals, families, and communities and threaten to overwhelm health systems [2]. Disease-related risk factors include age, genes (inherited risks), long-term health conditions, lifestyle factors, sex and gender, cognitive function, and so on. Korea is a “super-aged society,” in which over 20% of the total population is 65 years or older [3]. Mental health issues such as depression, loneliness, and suicide ideation have been reported to increase rapidly with soaring cognitive dysfunction among Korean elders. Meanwhile, the older adults have the highest poverty rate, and the suicide rate in South Korea is the highest among Organization for Economic Co-operation and Development countries [4]. This implies that the burden of psychological diseases in older Koreans is rapidly increasing, and the clinical approach alone cannot solve these problems. More innovative interventions should be included in the nonclinical approach to disease prevention and control for a healthier life [5].

Living alone or loneliness and a lack of social support are widely recognized risk factors for psychological disorders such as suicide, depression, and anxiety in later life [6]. These challenges are often exacerbated by life events like retirement or the loss of a loved one, which lead to smaller social networks and increased isolation [7]. The mental health of older individuals living alone is notably worse than that of those who cohabit, and those without spouses are at an even higher risk of depression [8]. During the COVID-19 pandemic, loneliness became more prevalent, with older adults being twice as likely to experience depressive symptoms compared to the prepandemic period, especially among those with lower socioeconomic status or existing health vulnerabilities [9-11]. These conditions not only increase the likelihood of depression and anxiety but also contribute to functional decline, disability, decreased life satisfaction, and even higher mortality rates [12-15]. The COVID-19 pandemic has highlighted the need for robust care support systems and high-quality life for the older adults, especially in addressing issues such as social isolation and loneliness.

Additionally, subjective health status, economic level, and literacy are crucial determinants of psychological health among the older adults. How older adults perceive their health often reflects their mental state, with positive perceptions being linked to better mental well-being and negative ones to poorer outcomes [16]. Economic conditions also play a pivotal role; older individuals with higher subjective economic levels and those receiving financial support from family members report higher levels of life satisfaction and lower risks of depression [17]. Notably, literacy significantly impacts mental health, particularly in rural older populations where low literacy or illiteracy is common. Poor literacy not only limits social participation but also reduces the effectiveness of interventions like social prescribing programs, hindering efforts to improve mental health outcomes in these communities [16]. Addressing these interconnected factors is essential for promoting better mental health among older adults.

Social prescribing originated in the United Kingdom in 2016 as an approach to connect patients or clients with nonclinical services in their local communities, addressing socioeconomic and psychosocial factors that influence health. The social prescribing model enables primary care professionals, particularly general doctors, to refer patients to various community-based activities and services, such as volunteering, gardening, and cooking, which voluntary and community organizations provide [18]. These services are designed to enhance the well-being of individuals, while simultaneously reducing the strain on conventional health care systems. The social prescribing structure has evolved over time. It generally consists of 3 key elements: the health care professional who makes the initial referral, community organizations offering support, and a link worker who facilitates the connection between the patient and the services [19]. The link worker works closely with the patient to identify their needs and goals, offering motivation and support throughout the care process. Recently, social prescribing has expanded internationally and is currently being developed in more than 30 countries and regions [20].

Social prescribing has been shown to benefit older adults [21,22]; however, given the diverse realities of aging, such as bereavement and cognitive decline, personalized interventions are essential [23]. Generic approaches often fail to meet individual needs, highlighting the importance of tailoring community care to specific situations and preferences. Integrating person-centered principles into social prescribing design can enhance intervention effectiveness by co-designing with individuals in ways that honor local culture and increase their sense of control, involvement, and engagement [24]. Around the world, social prescribing models vary but consistently reflect this emphasis on personalized care [25]. In the United Kingdom, social prescribing is integrated into the health care system, linking primary care professionals to community resources for social and psychological support that respect each person’s cultural and social context [26]. In Japan, social prescribing initiatives focus on mental health and social isolation, as highlighted in a recent study that implemented social prescribing practices in local settings to improve community mental health and well-being [27]. Research on Canada’s social prescribing in rural and underserved communities emphasizes the importance of local partnerships in providing social support and reducing isolation among older adults [28]. These diverse social prescribing approaches, though varying across countries, enhance older adults’ well-being by prioritizing person-centered care, fostering greater acceptance and engagement [29].

Adopting the UK’s concept at the beginning of 2019, Korea emerged as a notable example of a nongovernment-led program, with social prescribing being tested several times for older adults in a rural area [30]. This pilot project was led by a university and funded by the National School Research Foundation. Based on the special characteristics of the older adults in this rural area, the proportion of older people living alone who are depressed is relatively high, and a 10-week social prescribing program that includes musical storytelling, community gardening activities [31], and support groups has been developed [32]. Previous qualitative studies reported positive results in reduced depression, improved self-esteem, increased vitality through intergenerational interactions, and expanded social relationships [33]. Reduced loneliness, increased social engagement attitudes and self-esteem [34], and more active social support and life satisfaction were also observed during the COVID-19 pandemic pilot period [35,36].

Despite the considerably high prevalence of chronic conditions, social isolation, loneliness, and mental illness among Korean older adults (prior to the pandemic and exacerbated during this time), social prescribing has not yet been implemented on a national scale. Although there is a growing body of qualitative social prescribing research on a global scale, significant gaps remain in the literature (particularly in quantitative studies). Most notably, how to evaluate the implemented project model with more samples is the priority problem in each region. To address this gap, the present study explores the significant differences in social support, loneliness, depression, cognition, and life satisfaction among rural Korean older adults before and after receiving the pilot social prescribing project intervention, and assesses the effectiveness of the pilot project in which they participated. The findings contribute to the development of social prescribing initiatives that target specific needs, preferences, and expectations of older adults, ultimately enhancing their health, social engagement, and well-being.

## Methods

### Project Objectives

The pilot project was organized by Yonsei University to achieve two goals: (1) to reduce depression and loneliness in the older population in rural settings in Korea and (2) to help establish city-rural social capital and relationships among older people.

### Study Design

Participants were divided into experimental and control groups to evaluate the impact of a social prescribing program on depression, cognitive function, and loneliness among the older adults (April 2022‐ December 2022). The design used a nonequivalent control group pretest-posttest model. This approach allowed researchers to compare outcomes before and after the intervention in both the experimental (who received the social prescribing intervention) and control groups (who did not participate in the intervention).

The following are the key steps in the study design:

1. Preliminary survey: Before each intervention, a preliminary survey was conducted to select eligible participants based on set inclusion criteria, such as age, absence of severe depression (as measured by the short form Geriatric Depression Scale-Korean version, GDS-K), and capability for everyday communication.
2. Assignment to groups: Participants were assigned to either the experimental or control group based on their willingness to participate in the social prescribing program.
3. Intervention implementation: The experimental group participated in a 2-month social prescribing program that involved activities designed to reduce isolation and improve mental health.
4. Postintervention assessment: After completing the program, both groups underwent a posttest to assess changes in depression, cognitive function, and loneliness on the final intervention day.

### Intervention Programs

Music storytelling (Table 1), self-help groups, community gardening, Kalimba learning, flower tea classes, smartphone education, and COVID-19 prevention sessions were conducted. The related study protocol and research were also published in the previous studies [32,37].

**Table 1.** Intervention activities in the social prescribing program.

Intervention	Activities	Location	Time
Music storytelling	Rhythm for life Guide image music Movement improvisation circle	Community library	1 per week (90 min)
Self-help group (School of Hunjang^a^)	Healthy 100-year lecture: fruit Making natural soup for COVID-19 prevention Making masks for COVID-19 prevention Making natural diffuser Songwriting for the community	Community library	1 per week (60 min)
Community gardening	Gardening education in the spring and summer	Community library	Community garden 1 per week (30 min)
	Nursery plant (cherry tomato, sweet potato, corn, and pepper) Monitoring and managing community garden Harvesting	Wonju City Agricultural Center	Community garden 1 per week (30 min)
Kalimba class (hand-held musical instrument)	Learning, practice, and public performance	Community library	1 per week (60 min)
Flower tea class (with nutrition facts)	Learning and flower tea making Private selling	Community library	1 per week (60 min)
Smartphone education	Six-week smartphone education intervention for 65 and older people	Community library	1 per week (60 min)		
COVID-19 prevention session	Voluntary visit (per week)	—^b^	3 per week
	COVID-19 phone call (per week)	—	4 per week
	Education of healthy nutrition in COVID-19 pandemic	—	1 per week

a Hunjang: The teacher or headmaster of the Seodang was called the Hunjang.

b Not applicable.

- Music storytelling: Music therapy sessions were the main social program. The intervention was implemented as a multicomponent social prescribing package, with music storytelling as the core component and other activities serving complementary roles to support social engagement, peer support, and accessibility within the social prescribing framework. Rhythms, group dances, and singing sessions are examples of music therapy. Sessions took place every Thursday for a duration of 10 weeks. Moreover, the team encouraged the participants to exercise while listening to music. Examples include stretching, practicing deep breathing, and joining an indoor walking program (first, the program instructor demonstrated appropriate movements and positions so that participants could easily follow along) [32].
- Self-help groups: Participants gathered at the community library every Friday to make various products using their own hands. The participants made COVID-19 masks, dolls, and soaps. While working on their personal projects, the participants told each other old stories and taught each other how to make the products.
- Community gardening activities, Kalimba class, and flower tea class: Participants and volunteers planted corn, peppers, sweet potatoes, cabbage, and tomatoes during the farming program. Kalimba and flower tea classes were also added to improve older people’s hand activities and social contact.
- Smartphone education: Participants were divided into 6 groups to conduct the “smartphone education” session with education instructors and volunteer students. The education plan included basic smartphone operation methods to use apps most commonly used in Korea, such as Naver apps (information search platform) and KakaoTalk (mobile messaging app). Digital support in this study was intentionally designed as a low-threshold and supportive component rather than a technology-intensive intervention. Smartphone-based activities focused on basic functions and were embedded within face-to-face community programs to enhance accessibility and inclusivity among older rural adults. Additionally, every Friday, 2 instructors and a health major researcher shared feedback on the contents of the week’s education, adjusting the overall flow of education to be consistent with the difficulty level.

### Project Pilot Areas

The pilot areas for this research were Heung-eop-myeon and Munmak-eup in Wonju City, Gangwon Province, Korea. As of August 2022, Gangwon Province ranks fourth nationwide, with a high aging rate (Jeollanam-do 24.9%, Gyeongsangbuk-do 23.5%, Jeollabuk-do 22.9%, and Gangwon-do 22.4%). Both Heung-eop-myeon and Munmak-eup in Wonju City are experiencing high levels of aging with rates of 21.1% and 20.7%, respectively, indicating that both areas have become super-aged societies.

### Participant Recruitment

The town office and welfare center of Heungup Town provided a potential participant list and communication support. As detailed below, participants, who were being monitored by the local welfare center, were older than 65 years of age, had mild depression, provided informed consent, and lived within walking distance of the community library were selected for the pilot project:

1. The social welfare officer of the Heungup-myeon Administrative Welfare Center recommended the older participants (age ≥65 y and living alone) living in rural areas, Wonju City, Gangwon-do.
2. First participant selection: The researchers conducted the GDS-K test with the candidates to identify the range of depression and selected those who had mild or moderate symptoms (GDS-K score: 9‐18).
3. Second participant selection: The researchers selected older people who agreed to participate in the pilot and survey and had no difficulty with daily conversation and physical behavior.
4. Third participant selection (final): Public health doctors conducted an overall health diagnosis. Based on the results, the researchers selected the older adults who had no severe depression as the final participants in the pilot project.

### Program Participants Selection

Selection criteria for program participants: The program participants, consisting of experimental and control groups, were residents aged 65 years and older from Heung-eop-myeon and Munmak-eup in Wonju City, Gangwon Province. The other criteria were as follows:

1. The first criterion was that the participants had to be recommended by at least 1 local social worker, primary health care provider (doctor), or community health nurse to have no issues participating in the pilot project.
2. The second criterion was that participants must be capable of everyday communication without hearing or visual impairments.
3. The third criterion was that during the preliminary survey, participants must not have severe depression (normal-0‐9; mild depressives-10‐19; and severe depressives-20‐30), as determined by a score of 16 or lower on the GDS-K, a tool for detecting depression.

### Sample Size

The sample size for participants was determined using G*Power 3.1 (Erdfelder, Faul, & Buchner, 1996), with an effect size of 0.5, a power of 0.95, and a significance level of .05, resulting in a minimum sample size of 54. Participants were conveniently sampled from community libraries and senior welfare centers in Wonju City, totaling 294 individuals. Among them, 148 who wanted to participate in the intervention program were designated as the experimental group, and 146 who did not wish to participate were designated as the control group.

### Data Collection

Data were collected through one-on-one surveys by seven researchers, including 6 specializing in public health and 1 specializing in preventive medicine. The surveys were administered after explaining them to the participants, ensuring their understanding of the content, and obtaining their consent. The average survey time was approximately 15‐20 minutes per participant. The surveys conducted before and after the intervention included questions designed to measure sociodemographic indicators, depression, cognitive function, loneliness, social support, and life satisfaction. Data collected from these assessments were analyzed to evaluate the impact of the social prescribing intervention by comparing the preintervention and postintervention results between the experimental and control groups to determine the program’s effectiveness.

### Variables and Measurement

#### Selection of Study Variables

Building on previous research and the opinions of two local experts, this study selected age, sex, literacy, marital status, living alone status, religion, subjective economic level, and subjective health status as control variables. To measure the effectiveness of the pilot project, depression, social support, life satisfaction, cognitive function, and loneliness were selected as dependent variables.

#### Measurement of Study Variables

The short form GDS-K was used to measure depression among the participants. Higher scores indicated greater depression, with 15 items (15 scores) and a cutoff score of 8 [38]. The 8-item short version of the Perceived Social Support through Others Scale-8 estimated social support levels among these participants, and the highest score was 40 [39]. The Life Satisfaction Scale for Korean older adults was used to measure the index of their life satisfaction, which included 20 items and 3 scores [40]. Cognitive function was assessed using the Mini-Mental State Examination Korean Version (MMSE). For measuring loneliness, the shortened University of California, Los Angeles Loneliness Scale was used [41]. This scale consists of 8 items, with a total possible score of 32, where higher scores indicate greater levels of loneliness.

### Statistical Analysis

The collected data were analyzed using SPSS Statistics version 26.0 (IBM Corp), using the following methods:

First, sociodemographic characteristics, depression levels, social support, life satisfaction, cognitive function, and loneliness levels of the participants were analyzed using percentage and frequency analyses.

Second, to verify the homogeneity between the experimental and control groups, *t* tests and Chi-square tests were conducted for all measured variables (including sociodemographic indicators).

Third, paired and independent *t*-tests were conducted to identify significant differences in social support, loneliness, depression, cognitive function, and life satisfaction scores before and after the intervention.

Fourth, to explore significant changes in social support, loneliness, depression, cognitive function, and life satisfaction between the experimental and control groups, simple and multiple difference-in-differences (DID) analyses were performed.

Finally, to examine the direct and indirect relationships between social support, loneliness, and life satisfaction with depression and cognitive function among older individuals, structural equation modeling (SEM) using SPSS Amos (version 23.0, IBM Corp) was conducted. Goodness-of-fit was evaluated using the following indicators: adjusted goodness-of-fit index (≥0.80), comparative fit index (≥0.80), incremental fit index (>0.80) [42,43], and root mean square error of approximation (≤0.80) [44,45].

All statistical significance levels were set at *α*=.05, and all statistical tests were 2-tailed.

DID analysis is a statistical method that can be applied when there is an experimental group and a control group, and the time point before and after the intervention is divided. This method is widely used to evaluate the effectiveness of health care policies [46-48] and community-based health projects [49,50]. A control group was selected from the same area where the experimental group lived, and presurvey and postsurvey data were collected and analyzed for the same subject in both groups through the same questionnaire. Through this, regional and individual characteristics were controlled, and the effectiveness of the intervention was verified. The amount of change in the effect variable value in the control group from the amount of change in the effect variable value before and after the intervention of the experimental group was used to confirm the effect of the social prescription program intervention. In the United Kingdom, a study using the double-difference analysis was used to assess the effectiveness of social prescription programs for patients with type 2 diabetes [51].

### Ethical Considerations

The study was conducted in accordance with the Declaration of Helsinki. Before conducting the survey, the purpose of data collection and use was explained to the participants, and consent was obtained with the promise of maintaining anonymity regarding the use of the research data. Each participant received a small gift (10 $) after each session. All data were deidentified, stored securely, and accessible only to authorized research team members to ensure privacy and confidentiality. This study also received institutional review board approval from the Yonsei University Future Campus Bioethics Committee (1041849‐202206-SB-105-01).

## Results

### Participant Characteristics

Results in Table 2 demonstrate the key findings regarding the experimental and control groups’ sociodemographic characteristics and changes in the measured variables. The mean age of the 2 groups did not have a statistically significant difference (*P*=.60). A notable difference in gender distribution was found, with a higher proportion of women in the experimental group than in the control group (*P*<.001). Additionally, there were no statistically significant differences in living alone status (*P*=.57) and partner status (*P*=.25), indicating similar living conditions in both groups. Education levels demonstrated that approximately 116 (78.4%) had education and literacy, and 97 (65.5%) to 103 (70.5%) had their own religion in both groups (*P*=.41 and *P*=.36, respectively). Economic levels and subjective health status showed statistically significant differences, with the experimental group having fewer individuals at a high economic level (*P*<.001) and the experimental group reporting worse health (*P*<.001). There were statistically significant differences in social support and life satisfaction between the 2 groups.

**Table 2.** Social demographic characteristics of experimental group and control group participants.

Variables and categories	Experimental group (n=148)	Control group (n=146)	*t* test/chi-square (*df*)	*P* value
Age (y), mean (SD)	79.31 (6.796)	78.25 (6.344)	1.396 (292)^a^	.60
Sex, n (%)			35.971 (1,294)^b^	<.001
Male	8 (5.4)	48 (32.9)		
Female	140 (96.6)	98 (67.1)		
Live alone, n (%)			0.330 (1,294)^b^	.57
No	64 (43.2)	68 (46.6)		
Yes	84 (56.8)	78 (53.4)		
Partner, n (%)			1.349 (1,294)^b^	.25
No	89 (60.1)	78 (53.4)		
Yes	59 (39.9)	68 (46.6)		
Education, n (%)			0.675 (1,294)^b^	.41
No	32 (21.6)	26 (17.8)		
Yes	116 (78.4)	120 (82.2)		
Have religion, n (%)			0.847 (1,294)^b^	.36
No	51 (34.5)	43 (29.5)		
Yes	97 (65.5)	103 (70.5)		
Subjective economic level, n (%)			16.384 (2,294)^b^	<.001
High	6 (4.1)	22 (15.1)		
Average	64 (43.2)	59 (40.4)		
Low	78 (52.7)	65 (44.5)		
Subjective health, n (%)			13.119 (1,294)^b^	<.001
Bad	87 (58.8)	55 (37.7)		
Good	61 (41.2)	91 (62.3)		
Social support, mean (SD)	24.96 (8.660)	23.75 (6.765)	1.160 (226)^a^	.009
Loneliness, mean (SD)	20.75 (13.404)	23.82 (14.054)	–1.587 (200)^a^	.43
Depression, mean (SD)	11.68 (7.279)	10.17 (7.811)	1.937 (292)^a^	.54
Cognitive function, mean (SD)	25.39 (4.125)	24.92 (4.447)	0.934 (292)^a^	.08
Life satisfaction, mean (SD)	23.01 (10.448)	25.09 (8.504)	–1.673 (248)^a^	.004

a *t* test.

b Chi-square test.

### Results of Simple 2-Way ANOVA

Table 3 shows the results of a simple 2-way ANOVA of the experimental and control groups after the social prescription program intervention, including indicators of social support, loneliness, depression, cognitive function, and life satisfaction.

**Table 3.** Results of sample DID^a^ analysis.

Variables and group (n)	Participants (n)	Pretest (A), mean (SD)	Posttest (B), mean (SD)	Difference, B–A (*P* values)	DID analyses
Social support					
Experimental	112	22.90 (8.40)	27.02 (8.51)	4.12 (.02)	2.79
Control	122	23.59 (6.77)	23.92 (6.82)	1.33 (.79)	
Loneliness					
Experimental	100	21.39 (13.21)	20.11 (13.69)	−1.28 (.62)	−0.91
Control	94	24.13 (13.90)	23.50 (14.36)	−0.37 (.83)	
Depression					
Experimental	146	13.41 (7.18)	10.34 (7.01)	−3.07 (.009)	−3.69
Control	144	9.87 (7.45)	10.49 (8.22)	0.62 (.63)	
Cognitive function					
Experimental	145	25.15 (3.72)	25.62 (4.51)	0.47 (.49)	0.39
Control	144	24.88 (4.41)	24.96 (4.52)	0.08 (.92)	
Life satisfaction					
Experimental	123	21.27 (9.64)	24.75 (11.01)	3.48 (.049)	4.14
Control	144	25.24 (8.56)	24.90 (8.49)	−0.66 (.83)	

a DID: difference-in-differences.

**Figure 1.**
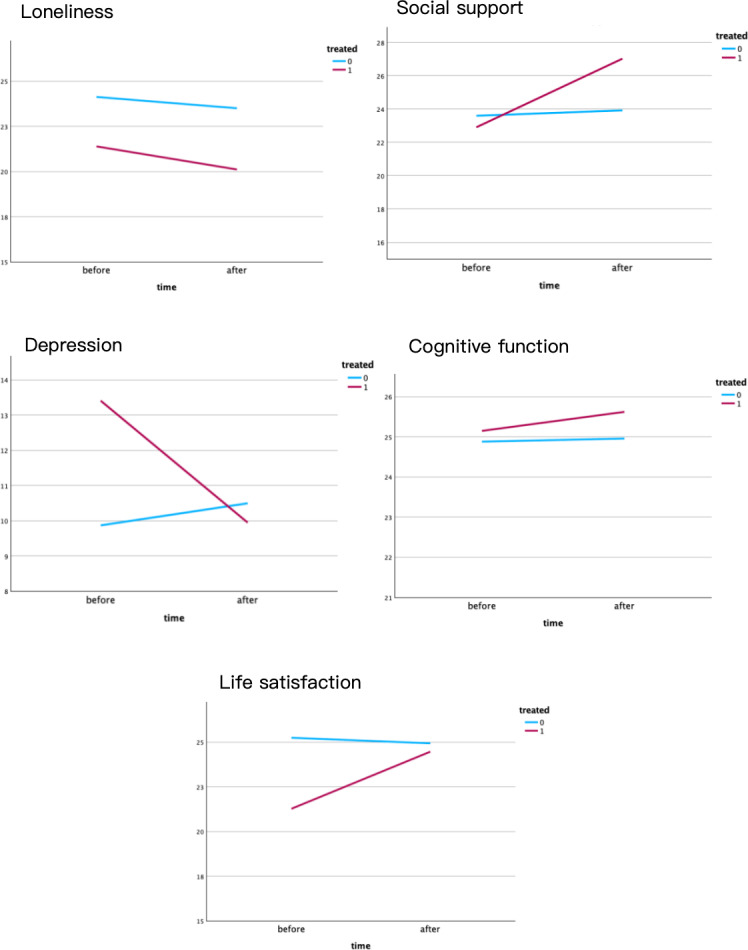
Experimental group-control group pre-post score for indicators. Treated 0: control group; treated 1: experimental group.

For social support, depression, and life satisfaction, the scores in the experimental group showed a statistically significant increase from a premeasure of 22.90, 13.41, and 21.27 to a post measure of 27.02 (*P*<.02), 10.34 (*P*=.009), and 24.75 (*P*=.049). Loneliness scores and cognitive function in both the experimental and control groups showed no statistically significant increase from the post test compared with the pretest. Depression in the experimental group demonstrated a statistically significant decrease, while the control group demonstrated increased depression scores without statistical significance (*P*=.63). Also, similar changes occurred in the control group in terms of social support (1.33) and life satisfaction (opposite, −0.66). Figure 1 shows the pre-post scores change graph in both groups.

### Associations Between Socioeconomic Factors and Social Support, Loneliness, Depression, Cognitive Function, and Life Satisfaction

The results of multiple DID analyses for socioeconomic factors and other indicators are shown in Table 4.

**Table 4.** Results of multiple difference-in-difference analysis.

Variables and categories	β				
	Social support (n=456)	Loneliness (n=404)	Depression (n=588)	Cognitive function (n=588)	Life satisfaction (n=500)
Time (reference: pretest)					
Post test	0.149	−0.158	0.978	−0.138	−0.743
Group (reference: control)					
Experimental	−1.102	−3.195	−2.605^a^	0.477	−3.056^a^
Interaction (time×group)	4.136^a^	−1.255	−4.121^b^	0.553	4.292^a^
Age	0.023	−0.376^a^	0.068	−0.168^c^	0.059
Gender (reference: male)					
Female	3.201^a^	−0.361	0.979	−0.971	1.735
Living alone (reference: yes)					
No	−0.802	−6.182	−0.558	2.014^a^	1.870
Partner (reference: no)					
Yes	1.144	0.264	0.796	2.085^a^	0.210
Literacy (reference: no)					
Yes	2.586^a^	−2.336	−2.296^a^	2.847^c^	2.344
Have religion (reference: no)					
Yes	0.446	−1.668	−2.165^a^	0.727	2.820^a^
Subjective health status (reference: bad)					
Good	3.856^c^	−0.734^a^	−5.165^c^	1.176^a^	7.439^c^
Subjective economic level (reference: low level)					
Middle level	−1.682	10.803	−3.562^a^	4.537^c^	5.029^a^
Upper level	−3.074	10.417	−3.430^a^	4.895^c^	5.300^a^
*R* ^2^	0.155	0.155	0.221	0.323	0.196
*F* test (*df*)	3.289 (12,443)	2.892 (12,392)	6.652 (12,575)	11.180 (12,575)	4.802 (12,487)
*P* value	<.001	<.001	<.001	<.001	<.001

a *P*<.05.

b *P*<.01.

c *P*<.001.

In the analysis of the relationship between these factors and depression scores, group and interaction variables were statistically significant. Comparing the experimental group, the control group showed a higher level of depression (*P*<.05); statistical significance was also found in the negative direction in the interaction variable (*β*=−4.121, *P*<.01), which confirmed that the intervention effect was found on the change in depression in the experimental group. Moreover, literacy (vs no literacy), having religion (vs no religion), good subjective health status (vs bad subjective health status), and high subjective economic level (vs middle or low subjective economic level) demonstrated a statistically significant negative effect on the depressive condition, indicating that older people who have literacy, specific religion, better subjective health, and better subjective economic level would have a lower level of depression. Similar results were found in the analysis of life satisfaction. However, no statistically significant relationship was found between literacy and life satisfaction.

Gender differences were apparent in social support, with female participants reporting higher perceived levels of social support than their male counterparts (*β*=3.201, *P*<.05) in the model of socioeconomic factors and social support. Additionally, social support scores seemed to have no relationship with subjective economic level. As a result of analyzing loneliness and cognitive function as an effect variable, neither the time variable, group variable, nor interaction variable were statistically significant. Surprisingly, statistically significant negative effects of age and subjective health status were reported in the loneliness analysis model; loneliness statistically significantly decreased as age increased (*β*=−.376, *P*<.05) with a better subjective health status (*β*=−.734, *P*<.05). However, for cognitive function, it was confirmed that younger persons who did not live alone and had a partner, literacy, better subjective health, and better subjective economic level had higher cognitive function scores.

### Impacts of Social Support, Loneliness, Cognition, and Depression on Life Satisfaction Among the Participants

**Figure 2.**
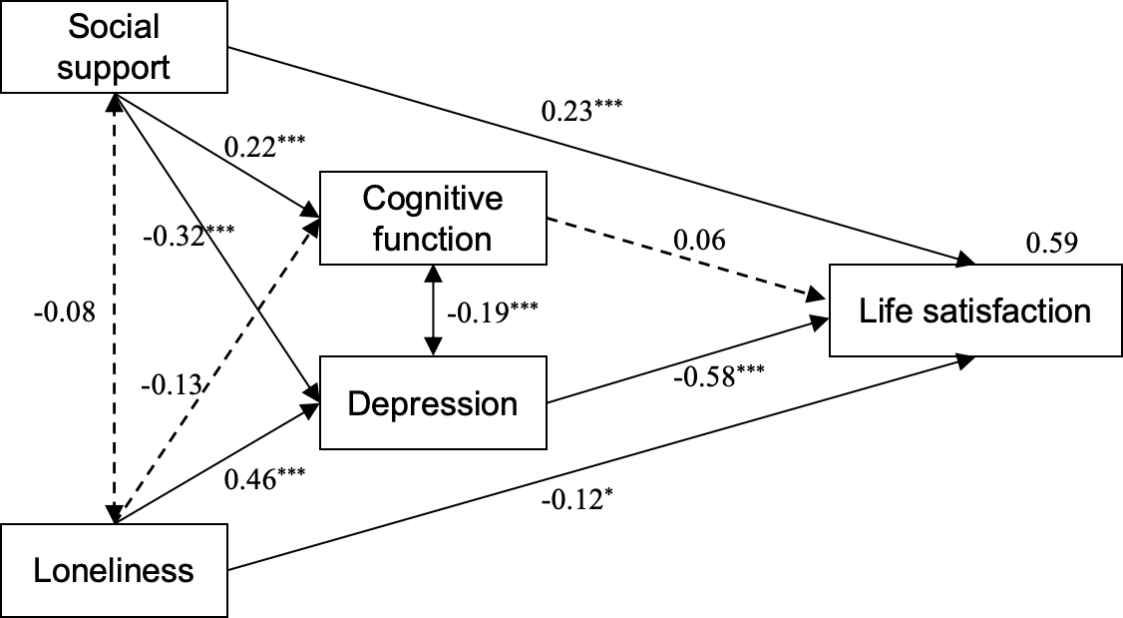
Structural equation modeling analysis of the association among social support, loneliness, cognitive function, depression, and life satisfaction. (*χ*^2^=1.50; *P*=.22; comparative fit index=0.99; incremental fit index=0.99; Tucker-Lewis index=0.97; root mean square error of approximation=0.041). Solid lines: significant paths; dotted lines: nonsignificant paths; *P<.05, ***P<.001.

SEM analysis (Figure 2) illustrates the association and standardized effects of social support, loneliness, cognition, and depression on life satisfaction among the participants. The level of social support positively affected cognitive function (*β*=.22) and life satisfaction (*β*=.23) and had a negative relationship with depression levels (*β*=−.32). Additionally, all associations between loneliness, depression, and life satisfaction were statistically significant: loneliness had a positive effect on depression (*β*=.46), and life satisfaction was negatively impacted by depression (*β*=−.58) and loneliness (*β*=−.12), which showed the mediating effect of depression. Surprisingly, no statistically significant relationship was found between loneliness and social support or cognitive function in this study.

Regarding the relationships between cognitive function, depression, and life satisfaction, a statistically significant negative correlation was demonstrated between cognitive scores and depression (*P*<.001), whereas the opposite was observed for the relationship between social support and cognition. Moreover, although there were both negative effects between social support and depression, loneliness and cognitive function, statistical significance was only reported in social support and depression (*β*=−.32). This indicated that depression was negatively impacted by social support, and that a higher level of social support would result in a lower depressive condition. Fit indices of the SEM model showed an overall goodness of fit for the model: χ^2^/df=1.50, root mean square error of approximation =0.041 (95% CI 0.000‐0.050; *P*=.02), and comparative fit index =0.99.

## Discussion

### Principal Results

This study assessed the effects of a social prescribing pilot project on the older adults in rural South Korea. The intervention aimed to alleviate depression and loneliness, while fostering cognitive function, social support, and life satisfaction. Consistent with real-world social prescribing practice, this pilot study focused on evaluating the overall effectiveness of an integrated, multicomponent intervention model rather than isolating the relative contribution of individual components. The results demonstrated statistically significant reductions in depression and improvements in social support and life satisfaction, alongside slight but nonsignificant improvements in cognitive function. SEM analysis further revealed a positive association between social support and life satisfaction, with depression and cognitive function acting as mediating factors, supporting the potential of social prescribing as a nonclinical, community-based approach to improving quality of life among rural older adults.

Research on social prescribing in the United Kingdom has highlighted its role as a valuable complement to clinical treatments for depression, with benefits for both physical and mental health [52,53]. A related study revealed that individuals who engaged more frequently with link workers showed greater improvements in anxiety and depression [54]. Consistent with this evidence, participants in our structured social prescribing program experienced significant reductions in depression and improvements in social support and life satisfaction. Similar positive effects have been observed in global social prescribing studies, particularly in reducing depression through community engagement and personalized support [55].

The differential outcomes observed in this study likely reflect varying responsiveness across outcome domains. Affective and evaluative indicators, such as depression and life satisfaction, appear more sensitive to short-term increases in social engagement and perceived support—core features of the intervention. In contrast, loneliness represents a more deeply rooted subjective experience that may be less responsive to change over a relatively short intervention period, particularly among older adults with long-standing social patterns in rural settings. Although cognitive function showed slight improvement, it did not reach a statistically significant association with the program intervention. This may be partly attributable to the relatively short intervention duration, which was sufficient to influence affective and psychosocial outcomes but may have been inadequate to produce detectable changes in global cognitive function [56]. In addition, cognitive function was assessed using the MMSE, a screening tool with limited sensitivity to short-term or domain-specific cognitive changes [57].

The study also highlighted the role of individual and contextual characteristics in shaping intervention outcomes. Factors such as age, gender, living arrangements, literacy, religion, subjective health, and economic status were associated with variations in social support, depression, cognitive function, and life satisfaction. In rural contexts characterized by long-term residence and stable social ties, older age was associated with lower reported loneliness, consistent with prior evidence suggesting greater emotional regulation, acceptance of social roles, and more selective social networks in later life [58-60]. Gender differences further indicated that women may benefit more readily from social support, aligning with research showing stronger community integration among older women in rural settings [61]. These findings suggest that future social prescribing programs may benefit from gender- and literacy-sensitive designs to enhance inclusivity and effectiveness [62,63].

Living arrangements and social resources also played an important role. Participants who did not live alone nor had a partner demonstrated higher cognitive function, underscoring the role of sustained social and emotional engagement in cognitive health [64], supporting previous studies linking partnership with cognitive resilience [65]. Literacy and religious affiliation were associated with lower depression and higher life satisfaction, highlighting the importance of culturally and cognitively accessible intervention materials and the protective role of spiritual and community support [63,66]. Better subjective health and economic conditions were consistently associated with lower depression and higher life satisfaction, likely reflecting reduced stress and enabling participation in meaningful social activities [25,67]. Conversely, reliance on basic pensions or combined financial and caregiving support from family members was linked to higher depressive symptoms, while informal family care was associated with greater life satisfaction compared with formal long-term or government-provided care [68-70].

The interaction effect between time and group demonstrated that the intervention’s structured support and tailored activities effectively influenced mental health trajectories, suggesting that social prescribing interventions may be particularly effective in the older population by providing structured and continuous social engagement and support. These positive shifts emphasize the cumulative benefits of social prescribing, as repeated engagement in meaningful and supportive activities over time appears to foster mental well-being and social connectedness among older participants [71]. It is also possible that the cognitive effects of social prescribing operate indirectly through sustained improvements in social support and depression, suggesting that more pronounced cognitive benefits may emerge with longer engagement and follow-up [56]. This outcome may reflect older participants’ established social skills, accumulated life experiences, or resilience, which may make them better equipped to benefit from social prescribing’s community-based support, especially in terms of incremental mental health [53].

The results also indicated key relationships among social support, loneliness, depression, cognitive function, and life satisfaction in the older population. Social support had a positive impact on both cognitive function and life satisfaction, while showing a negative association with depression. This finding suggests that social support may serve as a buffer against depressive symptoms, facilitating a more satisfying life experience and supporting cognitive health in older adults. These outcomes are consistent with existing research demonstrating that social support is protective against mental health challenges, helping maintain cognitive function and emotional well-being in aging populations [72,73]. Additionally, loneliness was positively associated with depression, and depression negatively affected life satisfaction, indicating that depression may mediate the relationship between loneliness and life satisfaction [74,75]. This aligns with research indicating that depression is a critical factor in understanding the impact of loneliness on overall well-being, underscoring the importance of addressing both social support and mental health through social prescribing interventions [76].

Moreover, the absence of statistically significant direct associations between loneliness and social support or cognitive function warrants careful interpretation. Loneliness reflects a subjective emotional experience, whereas social support represents perceived or available resources embedded within social structures [77]. In rural communities with relatively stable and enduring social networks, older adults may continue to engage in routine social interactions despite experiencing emotional loneliness, which may attenuate direct associations in short-term analyses [78]. Consistent with this interpretation, our SEM findings indicated that loneliness may primarily influence well-being indirectly through depressive symptoms rather than exerting direct effects on cognitive function or perceived social support. Cultural norms emphasizing emotional restraint and endurance among older adults in rural Korea may further contribute to the underreporting of loneliness, partially explaining the observed pattern [79].

These results further suggest that the cognitive benefits observed in this study are more directly attributable to enhanced social support than to reductions in loneliness per se. Social support appears to play a central role in mental health resilience, as higher levels of social support were associated with lower depression and greater life satisfaction [80]. By strengthening social connections and perceived support, social prescribing interventions may therefore mitigate the negative impact of depressive symptoms on overall well-being, even in the absence of immediate changes in loneliness or cognitive function. This pattern aligns with existing evidence showing that structured social interactions and supportive relationships foster resilience against mental decline and contribute to improved quality of life in later life [81,82].

Taken together, these findings suggest that the mental health benefits of social prescribing in rural older adults are driven primarily by enhanced social support and its downstream effects on depression and life satisfaction, rather than by immediate reductions in loneliness. This highlights social support as a central and actionable mechanism through which community-based, culturally embedded interventions can promote mental health resilience and quality of life in later life. At the same time, the findings underscore the importance of considering contextual and affective pathways—particularly the indirect role of loneliness—when interpreting intervention mechanisms and designing future longitudinal studies to capture longer-term cognitive and psychosocial effects.

### Limitations

This study had some limitations. First, the nonrandomized design and participation introduced potential selection bias. Although baseline equivalence was assessed and intervention effects were examined using DID analysis with covariate adjustment, residual confounding cannot be fully excluded. Participants in both the experimental and control groups may have had higher motivation for social engagement and more positive attitudes toward community activities, which could have influenced the observed outcomes. Second, as a real-world pilot study, it was challenging to fully implement all elements recommended by established intervention guidelines [83]. Third, the relatively small sample size and short intervention duration limit the generalizability of the findings and the ability to assess sustained or delayed effects, particularly with respect to cognitive outcomes. The use of the MMSE, while appropriate for screening, may have limited sensitivity to short-term cognitive changes in relatively high-functioning older adults. Future studies with larger samples, extended follow-up, and more sensitive or objective cognitive assessments are therefore warranted.

### Conclusions

This pilot study examined the effectiveness of a community-supported and digitally supported social prescribing program in improving mental health and well-being among older adults in rural South Korea. The findings demonstrated that such interventions can significantly reduce depression and enhance social support and life satisfaction, reinforcing the value of integrating community-based resources with culturally relevant digital tools in elder care. These results underscore the incorporation of social prescribing into broader community care strategies to address mental health disparities in underserved aging populations.

The program’s effectiveness was driven by culturally tailored activities—such as music sessions, flower tea making, and gardening—implemented through locally accessible and digital supports that promoted sustained engagement. By encouraging collaboration among health care professionals, local facilitators, and residents, social prescribing moves beyond traditional clinical models and toward inclusive, socially grounded care. The study underscores the importance of embedding social prescribing within broader community care and healthy aging strategies, with intermediary roles such as link workers serving as key connectors between digital and local community resources in sustaining and scaling such programs within national mental health and aging policy frameworks.

### Implications for Practice and Policy

The findings of this study offer timely and policy-relevant implications for enhancing mental health promotion among older adults in rural and underserved communities. From a sustainability and public health systems perspective, the results suggest that social prescribing interventions can be effectively embedded within broader healthy aging strategies when they leverage existing community infrastructure, such as community libraries, welfare centers, and local volunteer networks. By emphasizing nonclinical, preventive, and person-centered approaches, social prescribing aligns with ongoing shifts toward community-based mental health promotion for aging populations.

In practice and policy, the integration of digitally supported components—implemented as low-threshold, supportive tools embedded within face-to-face community activities—can improve accessibility and continuity of care without exacerbating digital exclusion. At the same time, the institutionalization of intermediary roles, such as link workers or community coordinators, is critical for sustaining and scaling social prescribing frameworks by facilitating needs assessment, navigation of community resources, and coordination between medical and nonclinical services. At the national level, these findings align with South Korea’s priorities to reduce social isolation and promote healthy aging, underscoring the importance of embedding social prescribing within existing mental health and aging policy frameworks and establishing stable funding and governance mechanisms to support sustainable, community-driven mental health promotion.

## Abbreviations

DID: difference-in-differences
GDS-K: Geriatric Depression Scale-Korean version
MMSE: Mini-Mental State Examination Korean version
SEM: Structural equation modeling
